# From phylogenomics to breeding: Can universal target capture probes be used in the development of SNP markers for kinship analysis?

**DOI:** 10.1002/aps3.11624

**Published:** 2024-11-12

**Authors:** Kedra M. Ousmael, Ole K. Hansen

**Affiliations:** ^1^ Department of Geosciences and Natural Resource Management University of Copenhagen Rolighedsvej 23, 1958 Frederiksberg C Denmark

**Keywords:** amplicon sequencing, Angiosperms353, relatedness analysis, SNP development, targeted sequencing

## Abstract

**Premise:**

Leveraging DNA markers, particularly single‐nucleotide polymorphisms (SNPs), in parentage analysis, sib‐ship reconstruction, and genomic relatedness analysis can enhance plant breeding efficiency. However, the limited availability of genomic information, confined to the most commonly used species, hinders the broader application of SNPs in species of lower economic interest (e.g., most tree species). We explored the possibility of using universal target capture probes, namely Angiosperms353, to identify SNPs and assess their effectiveness in genomic relatedness analysis.

**Methods:**

We tested the approach in 11 tree species, six of which had a half‐sib family structure. Variants were called within species, and genomic relatedness analysis was conducted in species with two or more families. Scalability via amplicon sequencing was tested by designing primers and testing them in silico.

**Results:**

Adequate SNPs for relatedness analysis were identified in all species. Relatedness values from Angiosperms353‐based SNPs highly correlated with those from thousands of genome‐wide DArTseq SNPs in *Cordia africana*, one of the species with a family structure. The in silico performance of designed primers demonstrated the potential for scaling up via amplicon sequencing.

**Discussion:**

Utilizing universal target capture probes for SNP identification can help overcome the limitations of genomic information availability, thereby enhancing the application of genomic markers in breeding plant species with lower economic interest.

Parentage analysis, pedigree confirmation, pairwise genetic relatedness quantification, and/or kinship estimation are some of the ways DNA markers can be utilized to improve the precision and efficiency of plant breeding. Each of these analyses requires good‐quality markers with Mendelian segregation, but the number of required markers is relatively small and they can be positioned everywhere in the genome if they are unlinked. In contrast, marker‐assisted selection requires multiple specifically identified candidate genes, and genomic selection requires a vast number of genome‐wide single‐nucleotide polymorphisms (SNPs). However, the identification of markers, regardless of their quantity or distribution in the genome, requires the availability of genomic information. The fundamental principle behind detecting SNPs, the most commonly used markers in recent years, is the comparison of one sequence with another, utilizing a single genome assembly as a baseline against which all other sequences are compared (Leggett and MacLean, 2014). However, the accessibility of genomic information is largely confined to widely used and therefore commercially valuable species. This limitation arises because reference genomes are typically constructed as part of extensive projects that involve long‐read sequencing or a combination of long‐ and short‐read sequences, requiring significant time and financial investment (Leggett and MacLean, 2014). For instance, tree species used on a smaller scale or for more specialized production that are consequently of less economic importance often lack the necessary attention and financial resources to undertake the generation of high‐quality reference genomes.

In the absence of genomic resources, reference‐free methods, such as genotyping‐by‐sequencing (GBS) (Elshire et al., 2011), restriction site–associated DNA sequencing (RADseq) (Baird et al., 2008), and DArTseq (https://www.diversityarrays.com), have been employed for variant detection in various plant species. In these approaches, SNPs are identified by aligning comparable sequences across samples (Eaton and Overcast, 2020). Although these methods may be deemed cost‐effective, they can become cost‐prohibitive when dealing with numerous plant species of minor economic interest. Challenges such as missing data problems, genotyping errors, reproducibility issues, and the need for high‐quality DNA further complicate the implementation of these approaches.

Other approaches exist for detecting SNPs in species lacking a reference genome. For example, amplicon‐based targeted sequencing is based on PCR amplification, which requires sequence information for designing amplicon sequencing primers. In contrast, hybridization capture‐based targeted sequencing, known as target capture via hybrid enrichment, employs complementary probes to capture specific genome regions. These target enrichment probes are often designed to target conserved genes across species and genera (Bignoniaceae, Fonseca et al., 2023; Fabaceae, Vatanparast et al., 2018; Orchidaceae, Eserman, et al., 2021) or even larger groups, such as mosses (Liu et al., 2019), ferns (Wolf et al., 2018), and flowering plants (Johnson et al., 2019). Depending on the targeted sequencing kit used, the sequencing of genomic libraries can yield thousands of DNA loci across various individuals and species (Larridon et al., 2020). However, these probes are designed to capture highly conserved genome regions and low‐copy orthologous loci, primarily used for generating sequence data in phylogenomic studies of non‐model organisms (Johnson et al., 2019; Fonseca et al., 2023).

Angiosperms353, being a universal targeted sequencing probe kit, offers the advantage of facilitating targeted sequencing in plant groups for which there are limited genetic resources (Chau et al., 2018). The probe set was developed utilizing multiple sequence alignments from over 600 angiosperm species, targeting 353 putatively single‐copy protein‐coding genes identified by the One Thousand Plant Transcriptomes Initiative (Johnson et al., 2019). In addition to capturing highly conserved regions for phylogenomic studies, Angiosperms353 also captures flanking non‐coding regions, making it possible to detect genetic variation within these target regions. This suggests that the Angiosperms353 target regions may be valuable for discerning within‐species relationships, such as kinship or genomic relatedness, among individuals. Recently, Slimp et al. (2021) tested the variation in Angiosperms353 genes, confirming that sufficient variation exists to calculate population genetics and demographic parameters.

In this study, we aimed to explore the feasibility of identifying an adequate number of SNPs in the Angiosperms353 genes and subsequently assess the utility of these SNPs in genomic relatedness analysis. This was done in multiple species simultaneously to demonstrate the cost‐effectiveness of utilizing universal target capture probes for marker development and to check for variation across multiple species, providing more evidence that sufficient variation (SNPs) can be found in the regions. Additionally, to ensure the applicability of the identified SNPs for genotyping a large number of individuals in a breeding program, we employed the sequences to design short amplicon sequencing primers. Even though we only used tree species in this study, we believe that the method can be applied to any angiosperm species. The use of universal probes for SNP identification across multiple species concurrently will substantially reduce the cost of SNP identification in non‐model species.

## METHODS

### Plant material

Eleven angiosperm tree species were included in this work, comprising nine sampled from field trials, plant collections, and nurseries in Denmark and two sampled from breeding seedling orchards (BSOs) in Ethiopia. The sources of the plant material used are briefly described below, and an overview is given in Table 1. Six of the 11 species were represented by one to four half‐sib families, each consisting of five individuals (Table 1).

**Table 1:** Taxonomy, sampling details, ploidy, and sampling site information for the 11 species in this study.

Clade	Order	Family	Species	No. of half‐sib families	No. of individuals	Ploidy	Sampling site*
Rosids	Fabales	Fabaceae	*Acacia senegal*	—	5	Mixed	Greenhouse UCPH
Rosids	Sapindales	Sapindaceae	*Acer velutinum*	1	5	Tetraploid	F472b – Greve
Rosids	Fagales	Betulaceae	*Alnus glutinosa*	2	10	Diploid	F.507 – Vendelholm
Rosids	Fagales	Fagaceae	*Castanea sativa*	—	5	Diploid	F.488 – Hørup
Asterids	Boraginales	Boraginaceae	*Cordia africana*	4	20	Diploid	BSO F3 Ethiopia
Rosids	Fagales	Fagaceae	*Fagus orientalis*	—	5	Diploid	CASPIAN nursery
Rosids	Fagales	Fagaceae	*Fagus sylvatica*	—	5	Diploid	Hjorthede nursery
Rosids	Fabales	Fabaceae	*Faidherbia albida*	4	20	Diploid	BSO F10 Ethiopia
Asterids	Lamiales	Oleaceae	*Olea europaea*	—	6	Diploid	Botanical Garden, etc.
Rosids	Rosales	Rosaceae	*Prunus avium*	1	5	Diploid	F.397 – Himmelev
Rosids	Fagales	Fagaceae	*Quercus castaneifolia*	2	10	Diploid	F472a – Greve
**Total**					**96**		

*Note*: BSO = breeding seedling orchard; UCPH = University of Copenhagen.

* See main text for details.

***Acacia senegal*** **(L.) Willd.**: Two diploid individuals from family DIA2B1 in the Diamenar progeny trial, one diploid individual from family DB23 in the Diery Biran Stand, and two tetraploid individuals from family NG19B1 in the Ngane progeny trial were sampled from seedlings grown in the greenhouse of the Department of Plant and Environmental Sciences, University of Copenhagen (UCPH), Denmark. The trials are located in Senegal.

***Acer velutinum*** **Boiss.**: In the combined species and provenance trial F472b, established under the auspices of the CASPIAN project in 2019 (CASPIAN = The Caspian forests of Iran: A gene pool for the adaptation of European forests?), five individuals were sampled from one half‐sib family (AvGoL9) originating from the provenance Golestan Low in northern Iran.

***Alnus glutinosa*** **(L.) Gaertn**.: In the progeny trial F.507 – Vendelholm, among progeny from the best‐appearing trees chosen in Danish populations, five individuals were sampled from each of two half‐sib families originating from populations Meilgaard (family RAV4) and Rydhave Skove (family RYD7).

***Castanea sativa*** **Mill.**: Five trees with origins in a certified seed stand, F.859 Svenstrup in the provenance trial F.488 – Hørup (IGN no. 16), were sampled for this species.

***Cordia africana*** **Lam.**: The source of *C*. *africana* material in this study was the BSO F3 located on the International Livestock Research Institute campus in Addis Ababa, Ethiopia (9°0′50″N, 38°48′55″E). The BSO comprises 18 bulk collections and 53 families from three provenances: Adwa, Harar, and North Bench (Ousmael et al., 2024). We included two families with five individuals each from the North Bench provenance and one family each from Adwa and Harar, with five individuals per family.

***Fagus orientalis*** **Lipsky**: Five seedlings from the Danish nursery Hjorthede, produced with seeds from the Mazandaran Medium and Gilan Medium provenances in northern Iran, which were imported directly under the auspices of the CASPIAN project.

***Fagus sylvatica*** **L.**: Five seedlings from the Danish nursery Hjorthede, produced with seeds from the Danish seed stand F.596 DTU, with material originating from Sihlwald near Zurich, Switzerland, were obtained.

***Faidherbia albida*** **(Delile) A.Chev.**: The materials used in this study were obtained from the BSO F10, established by the Provision of Adequate Tree Seed Portfolios (PATSPO) in Mojo, Ethiopia (8°38′34″N, 39°06′41″E). In the BSO, there were 10 bulk seed collections and 64 families representing four provenances: Abrha Atsbha, Enfranz, Hawassa, and Jinka. One family was sampled from each of these provenances, with five individuals per family.

***Olea europaea*** **L.**: Two *Olea europaea* subsp. *europaea* and one *Olea europaea* subsp. *cuspidata* were obtained from the UCPH Botanical Garden, plus two individuals from the plant collections at the Frederiksberg Campus of UCPH and one seedling of a commercial variety of subsp. *europaea* bought from a builder's merchant also selling garden plants.

***Prunus avium*** **(L.) L.**: Five trees from the half‐sib family S21K916611 of the Knutstorp provenance in progeny trial F.397 Himmelev were used for this species.

***Quercus castaneifolia*** **C.A.Mey.**: In the combined species and provenance trial F472a ‐ Greve, established under the auspices of the CASPIAN project in 2019, five individuals were sampled from each of two half‐sib families (family QcMaL1 and family QcGoM2) originating from the provenances Mazandaran Low and Golestan Medium in northern Iran.

For seven of the nine species sampled within Denmark (except *O*. *europaea* and *A. senegal*), dormant buds were collected for DNA extraction, while leaf samples were used for the remaining four species. The buds were stored at −20°C, while the leaves were dried with silica gel. DNA was extracted using the DNeasy Plant Mini Kit (Qiagen, Hilden, Germany).

### Library preparation, target capture, and sequencing

DNA samples were used as input for a custom capture library preparation protocol. First, whole‐genome libraries were constructed using Illumina DNA Prep (San Diego, California, USA), strictly following the manufacturer's instructions. Libraries were dual‐indexed to allow for post‐sequencing de‐multiplexing. Libraries were purified twice using Mag‐Bind RxnPure Plus magnetic beads (Omega Biotek, Norcross, Georgia, USA), following the manufacturer's instructions.

Following preparation, libraries were pooled in equimolar amounts according to the pooling scheme specified in Appendix S1 (see Supporting Information with this article). Then, the myBaits Expert‐Plant‐Angiosperms353 probe kit (Daicel Arbor Biosciences, Ann Arbor, Michigan, USA) was used to hybridize specific regions of the genome to biotinylated probes, which were then captured with streptavidin‐coated magnetic beads. The captured library pools were quantified using the Qubit dsDNA HS assay (Thermo Fisher Scientific, Waltham, Massachusetts, USA) and pooled in equimolar amounts into a single final pool. The fragment size distribution and concentration of the libraries were checked using an Agilent 2100 Bioanalyzer (using the Agilent HS DNA Kit) (Santa Clara, California, USA). The pool was sequenced on a fraction (one‐quarter) of a MiSeq PE300 flow cell (Illumina).

### Data preprocessing and recovery of Angiosperms353 genes

FastQC (Andrews, 2010) was used to obtain an overview of the quality status of the raw sequencing reads per sample. Subsequently, individual FastQC reports were aggregated into an overall quality status report using MultiQC (Ewels et al., 2016). The raw reads were processed with FastP (Chen et al., 2018) to eliminate adapter sequences, G‐homopolymers, and reads with an average Q score below 20. Angiosperms353 genes were then retrieved from the preprocessed reads using the HybPiper pipeline (Johnson et al., 2016). In this recovery process, we utilized the mega353 target file (McLay et al., 2021) instead of the original Angiosperms353 target file. The mega353 target file represents an expanded version of the Angiosperms353 file with increased phylogenetic density, thereby containing more representative sequences per gene. HybPiper generated exon sequences corresponding to the target genes and supercontigs that contain exons and non‐coding regions assembled together.

### Generating reference sequences

**Figure 1:**
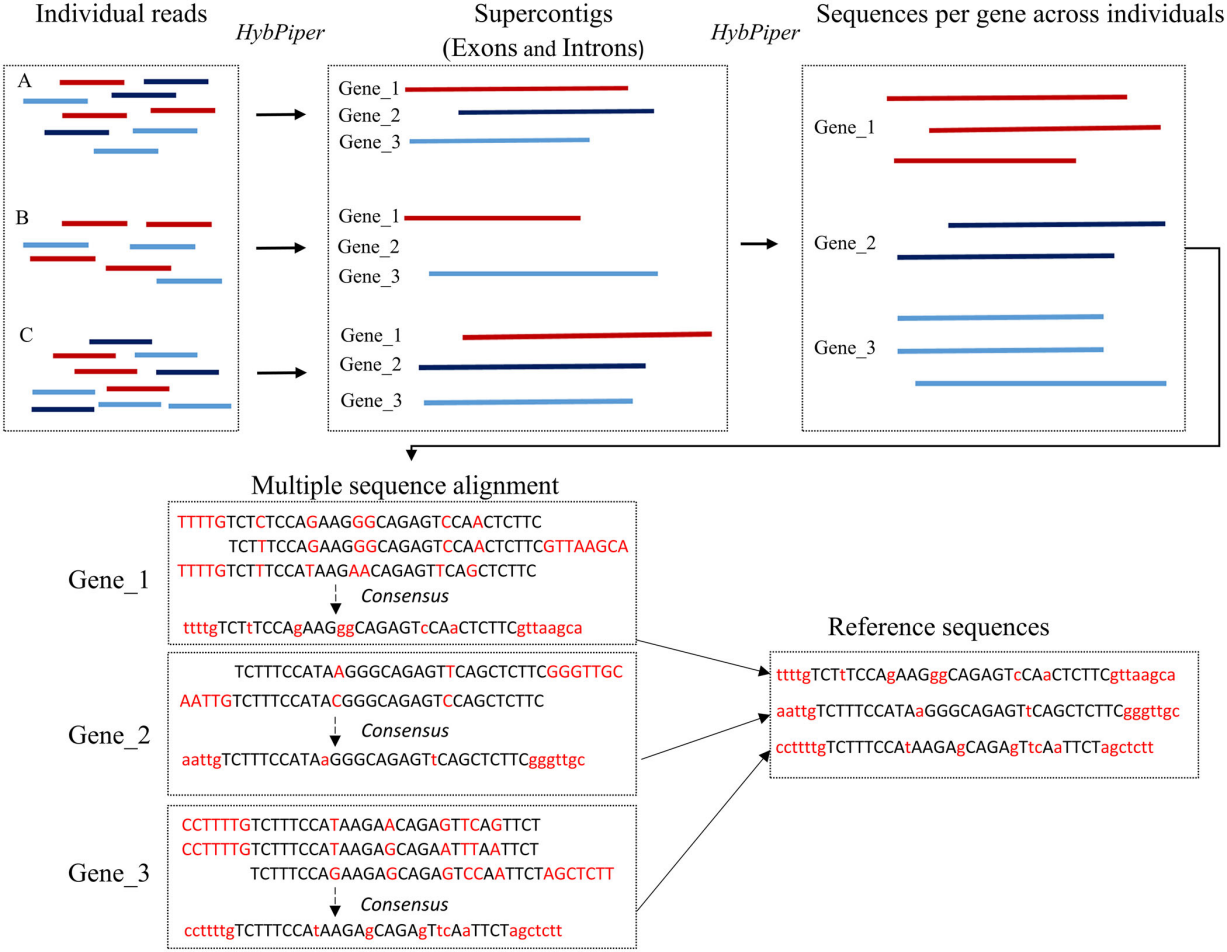
Schematic illustration of the workflow used to generate reference sequences.

To create references for each species, supercontigs were consolidated per gene using HybPiper's *retrieve_sequences* command. Subsequently, multiple sequence alignments were conducted for each recovered gene utilizing MAFFT (Katoh et al., 2009). Consensus sequences for each gene were then extracted from multiple sequence alignments using EMBOSS (Rice et al., 2000). The reference sequences were composed of consensus sequences from all recovered genes. The workflow employed to generate the reference sequences is illustrated in Figure 1.

### SNP identification and filtering

To identify SNPs, the raw sequencing reads were aligned to the generated reference sequences using Bowtie2 (Langmead and Salzberg, 2012). Subsequently, duplicates (GATK MarkDuplicates; https://broadinstitute.github.io/picard/) were marked before variant calling using BCFtools mpileup (Li, 2011). For *A*. *senegal* and *A*. *velutinum*, the two species with polyploid individuals, FreeBayes (Garrison and Marth, 2012) was used to identify the variants. Three of the *A*. *senegal* individuals were diploids, while the remaining two were tetraploids; therefore, SNPs were called separately for diploids and tetraploids. VCFtools (Danecek et al., 2011), vcflib (Garrison et al., 2022), and BCFtools (Danecek et al., 2021) were used to filter the raw variants. vcflib and BCFtools were used to filter *A*. *senegal* and *A*. *velutinum* variants, as VCFtools did not support polyploidy. Initially, indels and multi‐allelic SNPs were removed from the raw variants. The raw SNPs were then filtered based on minor allele frequency (MAF), where all non‐polymorphic loci were removed from species represented by only five individuals, and all loci with MAF < 0.05 were removed from the rest of the species. Additionally, considering the limited number of individuals (five) in some species, we chose to exclude SNPs with missing values. SNPs in species with ≥10 individuals were filtered for missing values <0.1. The SNPs underwent further filtering for genotype quality (GQ), mapping quality (MapQ), minimum depth (minDP), and maximum depth (maxDP). The filtered SNPs had a GQ > 16, MapQ > 40, minDP equal to half of the mean of the depth distribution, and maxDP equal to twice the mean of the depth distribution.

### Kinship analysis

To assess the utility of the identified SNPs for kinship analysis, we conducted genomic relatedness analysis in four species with more than one family sampled using the AGHmatrix R package (Amadeu et al., 2023), implementing a method by VanRaden (2008). Similar relatedness values were obtained using Plink (Purcell et al., 2007) and VCFtools, and the results from the AGHmatrix were used for downstream analysis. First, principal component analysis was carried out to visualize the grouping of families in the four species. The potential of the identified SNPs was then assessed by comparing the genomic relatedness obtained from the SNPs to the expected additive genetic relationships among half‐sib individuals (genetic correlation = 0.25). In addition, the 20 *C*. *africana* individuals in this study were previously genotyped by 3325 (after filtering for similar criteria) DArTseq SNPs (Ousmael et al., 2024), providing an opportunity to validate relatedness values obtained using information from a large number of genome‐wide SNPs in terms of resolving different types of relationships. This was performed by computing the correlation coefficient of the relatedness values obtained using the two sets of SNPs and by analyzing the relative grouping of the families via a combined heatmap of genomic relatedness values.

### Primer design and in silico validation

To test the application of the developed SNPs for large‐scale genotyping, we designed amplicon sequencing primers based on the generated Angiosperms353‐based reference sequences flanking the identified SNP loci. A total of 300 primer pairs with amplicon sizes of 100–150 bp (for randomly selected SNPs, while considering the distance between them) were designed for *F*. *albida* and *A*. *glutinosa* using Primer3 (Untergasser et al., 2012). Amplification success was tested in silico using EMBOSS *primersearch* with the reference genomes of *F. albida* (Chang et al., 2019) and *A. glutinosa* (Griesmann et al., 2018).

The success rate is reported in terms of the proportion of primers with amplimers (in silico–generated amplicons) and the number of amplimers per primer pair as a measure of specificity. The number of amplimers per primer pair could also be useful for testing the single‐copy nature of the target region. In addition, the fact that SNPs are identified in the conserved regions of the genome might allow for the design of primers that can work for more than a single species. However, the utility of a primer designed for one species in another depends on the presence of variations in the target region of the new species. In this study, we tested the utility of primers designed for *A*. *glutinosa* in the amplicon sequencing of the related species *Alnus rubra* Bong. The reference genome of *A*. *rubra* generated by Hixson et al. (2023) was used.

## RESULTS

### Gene recovery

**Figure 2:**
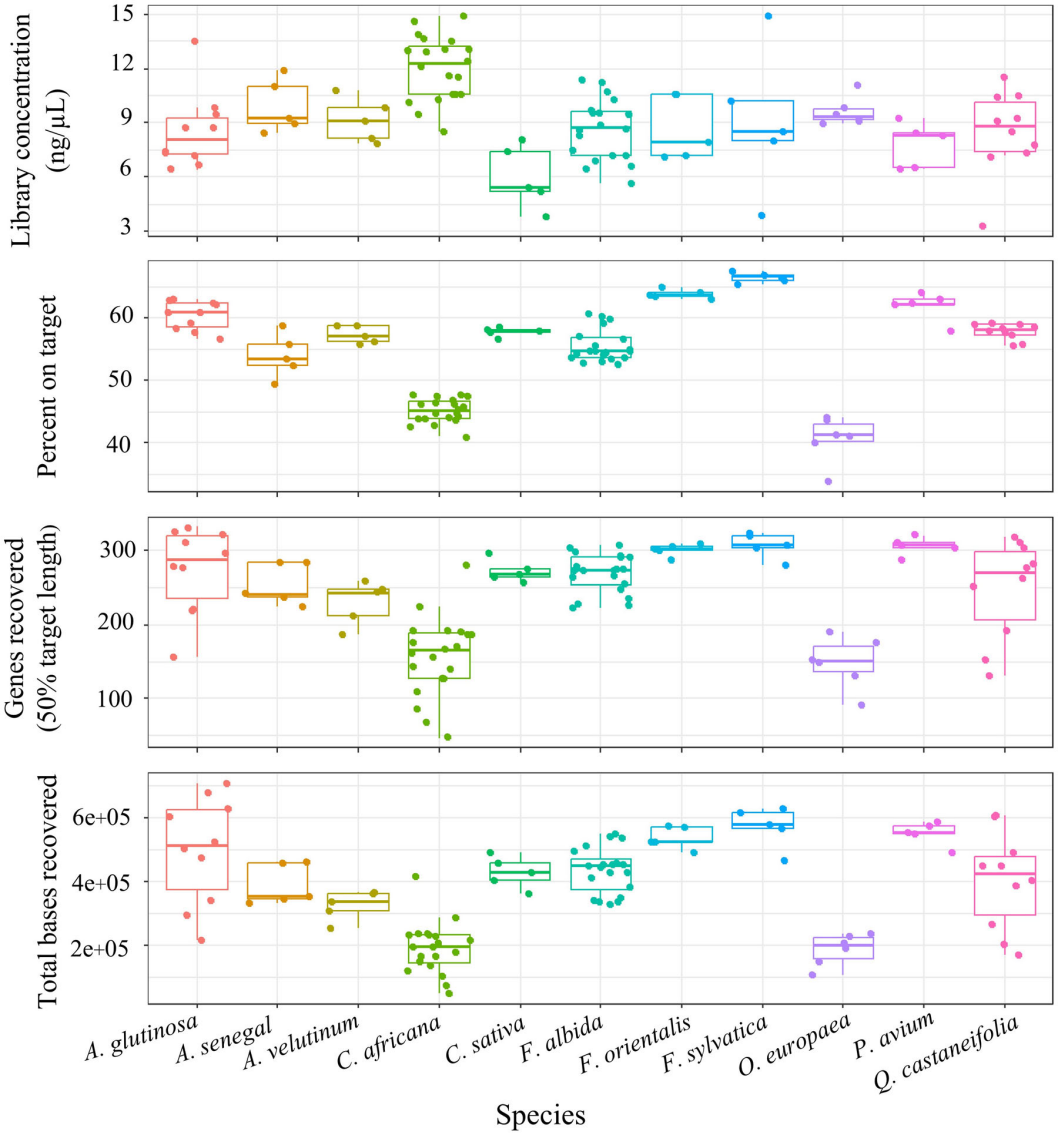
Distribution of genomic library concentration (ng/µL), enrichment efficiency (percent on target), gene recovery (genes recovered ≥50% of target length), and total bases recovered, including introns grouped by species.

The number of raw reads per sample varied from 34,436 to 142,928. After quality filtering, the number of reads ranged from 31,678 to 133,812. The number of genes with sequences ranged from 59 (in *C*. *africana* individuals) to 332 (in *A*. *glutinosa* individuals), with an average of 244. The enrichment efficiency, i.e., the percentage of reads mapped to the target, ranged from 33.8 to 67.5. Despite substantial variation among species, gene recovery proved successful for all individuals, with between 48 and 331 genes having at least 50% of the target region recovered (mean: 239) (Figure 2). See Appendix S2 for details on target recovery.

The library concentration appeared to have a negative impact on target enrichment efficiency (*r* = −0.43, *P* < 0.001). However, further investigation revealed that this negative correlation was primarily influenced by the confounding effect of species. *Cordia africana* and *O*. *europaea*, the species with the highest library concentrations (means of 12.03 ± 1.77 and 9.62 ± 0.79, respectively), exhibited the lowest target capture efficiency (Figure 2). Conversely, *C*. *sativa*, the species with the lowest mean library concentration (5.98 ± 1.74), demonstrated a target capture efficiency (57.82 ± 0.69) higher than the overall mean (54.68 ± 7.48). Examining the correlation between library concentration and target efficiency within species, we found only a moderate positive correlation (*r* = 0.49, *P* < 0.05) in *C*. *africana*. Overall, individuals belonging to the same species displayed target capture efficiencies on the same level, regardless of whether they were in the same hybridization pool. This shows that while target efficiency varies from species to species, there is less variation among individuals.

Among the analyzed individuals, 70.9% exhibited a total supercontig length exceeding the coding target region of Angiosperms353 (260 kbp; Johnson et al., 2019). Individual total supercontig lengths ranged from 51.4 (in a *C*. *africana* individual) to 711.4 kbp (in an *A*. *glutinosa* individual) (Appendix S2). The total supercontig length by species mirrored the patterns observed in target efficiency and gene recovery (Figure 2). *Fagus sylvatica* had the highest mean total supercontig length (573.5 ± 64.9 kbp), while *O*. *europaea* had the lowest (187.8 ± 51.0 kbp). Six of the 11 species had an individual supercontig (i.e., from a single gene) length exceeding 6000 bp, with *Q*. *castanaefolia* having the maximum individual supercontig retrieved (9580 bp) (Appendix S3). Only *C*. *africana* and *O*. *europaea* had a maximum supercontig length of slightly under 5000 bp.

### Variation within the Angiosperms353 target region

The number of raw SNPs identified in the 11 species ranged from 1040 in *P*. *avium* to 21,756 in *A*. *velutinum* (Table 2). The remaining species had more than 1500 raw SNPs identified, except for *O*. *europaea*, which had 1286. After filtering, *A*. *velutinum* had the lowest number of SNPs (182), while *F*. *albida* had the highest number (1115) (Table 2).

**Table 2:** Number of identified single‐nucleotide polymorphisms (SNPs).

Species	Raw SNPs	Filtered SNPs
*Acacia senegal* ^d^	6313	333
*Acacia senegal* ^t^	14,025	513
*Acer velutinum*	21,756	182
*Alnus glutinosa*	3712	1074
*Castanea sativa*	1759	512
*Cordia africana*	2276	237
*Fagus orientalis*	2747	918
*Fagus sylvatica*	2820	874
*Faidherbia albida*	5010	1115
*Olea europaea*	1286	275
*Prunus avium*	1040	321
*Quercus castaneifolia*	3554	918

d = diploid; t = tetraploid.

The SNPs in *A*. *glutinosa*, *Q*. *castaneifolia*, and *F*. *albida* required further filtration based on MAF for improved relatedness results. In *A*. *glutinosa* and *Q*. *castaneifolia*, SNPs with MAF <0.2 were removed, while in *F*. *albida*, those with MAF <0.1 were excluded. Additionally, loci with a heterozygosity >0.5 (the maximum theoretically expected in loci with two alleles) were removed from *A*. *glutinosa* and *Q*. *castaneifolia*. The number of SNPs used for downstream analysis in *A*. *glutinosa*, *Q*. *castaneifolia*, and *F*. *albida* was 349, 269, and 667, respectively.

### Clustering of half‐sib families

**Figure 3:**
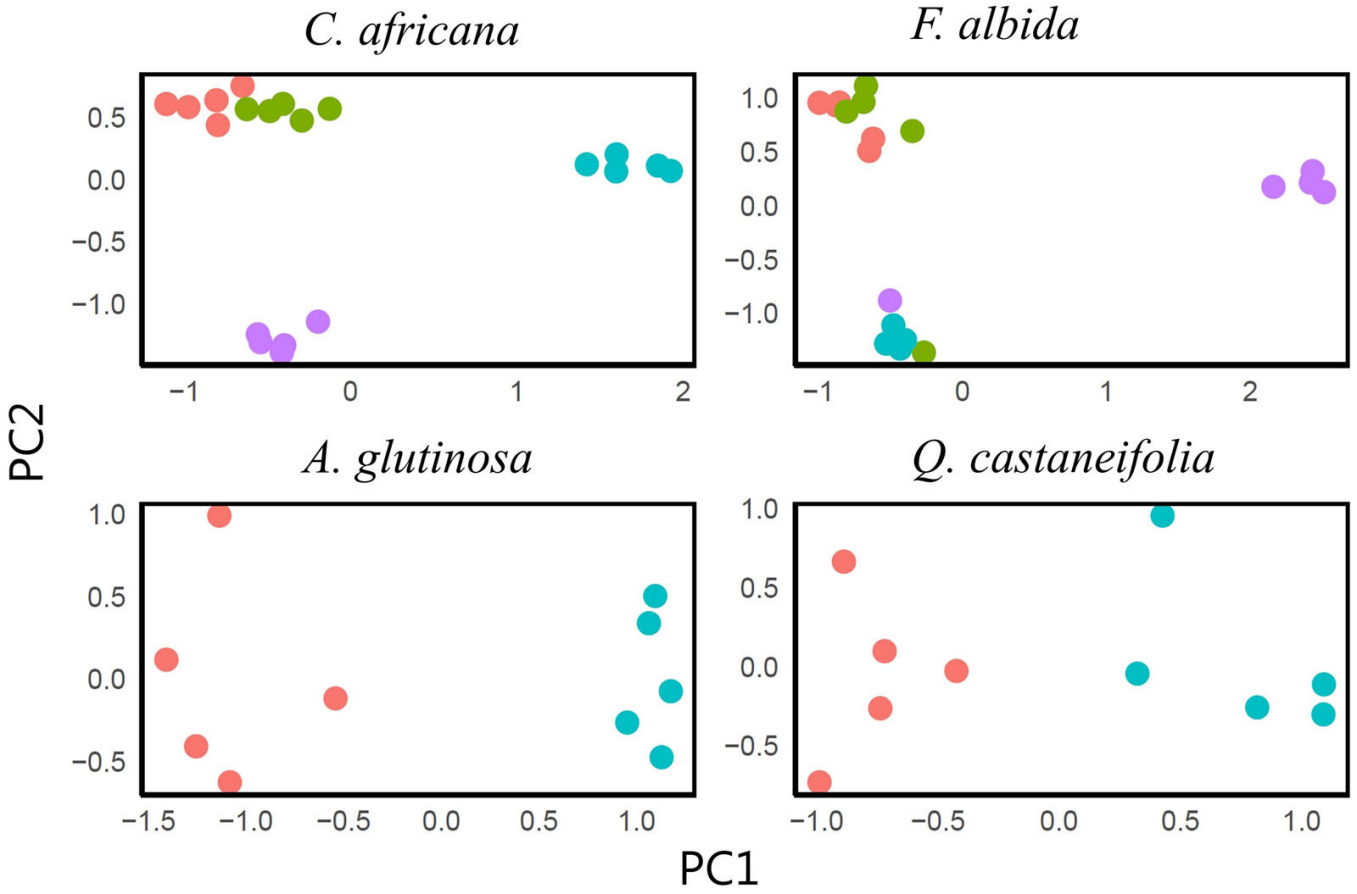
Clustering of half‐sib families (according to pedigree information) using principal component analysis of relatedness values from filtered SNPs identified within the target region of the Angiosperms353 probe in four species (*Cordia africana*, *Faidherbia albida*, *Alnus glutinosa*, and *Quercus castaneifolia*). Families are represented by different colors.

Principal component analysis revealed the grouping of most individuals from the same half‐sib families in the four analyzed species (Figure 3). The overall grouping exhibited 95% similarity with the expected grouping according to pedigree information in *C*. *africana* and 85% similarity in *F*. *albida*. However, the grouping of some families appeared to be more distinct than others. *Alnus glutinosa* and *Q*. *castaneifolia* demonstrated a grouping that was 100% similar to the expected grouping, but the clusters appeared less tight (Figure 3).

### Pairwise genomic relatedness

The mean pairwise realized genomic relatedness was computed for each family before and after pedigree correction. Correction involved two approaches: exclusion of individuals with low relatedness to all members within the species and reassignment of individuals to new families when they showed connections to individuals in other families. In *F*. *albida*, three individuals were reassigned to a different family. For *A*. *glutinosa*, a single individual was removed, and no correction was applied to *C. africana* and *Q*. *castaneifolia*.

Among the four species studied, *C*. *africana* exhibited a consistent relatedness level more in line with pedigree information (compared to the other species evaluated), with mean family relatedness in the four families ranging from 0.19 to 0.48 without any pedigree correction (Table 3). In *F*. *albida*, the families displayed varying genomic relatedness levels. Notably, family S74 showed a substantial increase in mean relatedness after correction (from 0.43 to 0.89), primarily due to the removal of an unrelated individual (related to another family), while other families maintained similar relatedness levels before and after correction. Conversely, *A*. *glutinosa* families showed relatively moderate genomic relatedness levels, with family RAV4 exhibiting an increase in mean relatedness after pedigree correction. In *Q*. *castaneifolia*, families demonstrated the lowest mean relatedness, comparable to unrelated individuals, where *r* = 0 (Table 3).

**Table 3:** Mean family pairwise realized genomic relatedness before and after pedigree correction.

Species	Family	Mean *r* ± SD BPC	Mean *r* ± SD APC
*Faidherbia albida*	S30	0.25 ± 0.13	0.25 ± 0.11
	S40	0.08 ± 0.28	0.38 ± 0.06
	S50	0.30 ± 0.09	0.28 ± 0.09
	S74	0.43 ± 0.60	0.89 ± 0.06
*Cordia africana*	S35	0.29 ± 0.13	0.29 ± 0.13
	S41	0.19 ± 0.07	0.19 ± 0.07
	S57	0.48 ± 0.10	0.48 ± 0.10
	S99	0.42 ± 0.07	0.42 ± 0.07
*Alnus glutinosa*	RAV4	0.14 ± 0.12	0.19 ± 0.13
	RYD7	0.16 ± 0.06	0.16 ± 0.06
*Quercus castaneifolia*	QcMaL1	0.02 ± 0.04	0.02 ± 0.04
	QcGoM2	0.02 ± 0.11	0.02 ± 0.11

*Note*: *r* = relatedness; SD = standard deviation; BPC = before pedigree correction; APC = after pedigree correction.

### Performance of Angiosperms353 compared to DArTseq SNPs

**Figure 4:**
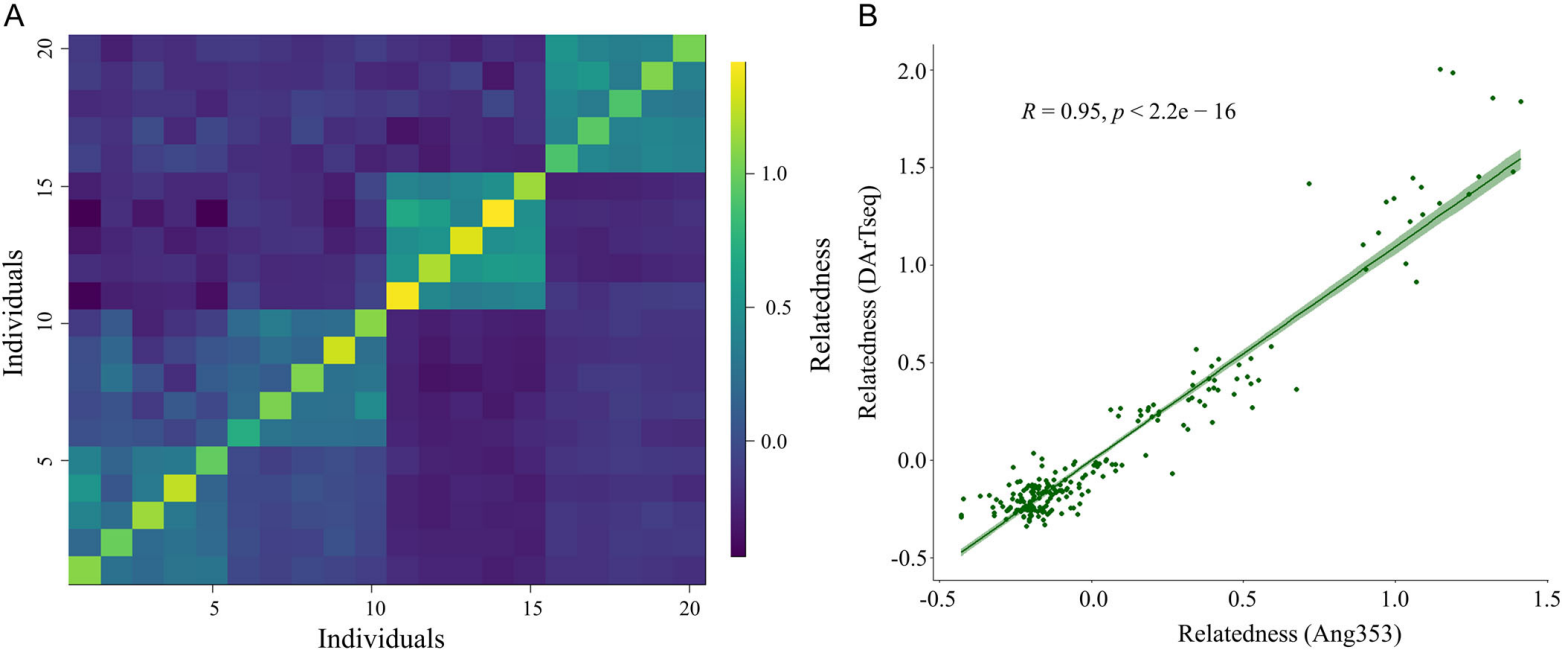
Comparison of genomic relatedness computed with SNPs identified based on Angiosperms353 (237 SNPs) and DArTseq SNPs (3325 SNPs) (Ousmael et al., 2024) in 20 individuals belonging to *Cordia africana*. (A) A heatmap showing pairwise genomic relatedness based on the Angiosperms353‐based SNPs in the upper diagonal and the DArTseq SNPs in the lower diagonal. The first two rectangles (diagonally from left to right: individuals 1–10) represent two families belonging to the same provenance, while the following two rectangles (individuals 11–15 and 16–20) represent two families, each representing a distinct provenance. (B) A graph showing the correlation between the genomic relatedness values computed with the two sets of SNPs.

The genomic relatedness analysis in *C*. *africana* using DArTseq and the Angiosperms353‐based SNPs grouped individuals into their respective families and showed relatedness between families belonging to the same provenance (Figure 4A). Moreover, the pairwise relatedness values for all types of relatedness, including self, computed using the two sets of markers showed a high correlation (*R* = 0.95; Figure 4B).

### Potential for scaling up

In both *A*. *glutinosa* and *F*. *albida*, 98.7% of the tested primer pairs resulted in amplimers in silico when a mismatch of 10% was allowed between the primer and template. The success rate decreased to 87.3% and 88.7%, respectively, when no mismatches were allowed (Table 4). In addition, the specificity of primer pairs, indicating the presence of a single target region in the genome (an indicator of the target region being a single copy), was evaluated. When considering the specificity of the primer pairs, the number of successful primers decreased in both scenarios, with and without allowing for mismatches. Specifically, 85.3% of *A*. *glutinosa* primers and 87% of *F*. *albida* primers generated single‐target amplimers. Furthermore, 57% of the tested *A*. *glutinosa* primers were transferable to *A*. *rubra* with no mismatch allowed, producing specific (single)‐target amplimers.

**Table 4:** Proportion of primers with amplimers and proportion with only a single amplimer per primer pair in two scenarios: one allowing for a mismatch between the primer and template and the other without such an allowance.

Species	10% mismatch		No mismatch	
	With amplimers (%)	With only 1 amplimer (%)	With amplimers (%)	With only 1 amplimer (%)
*Alnus glutinosa*	98.7	93	87.3	85.3
*Alnus rubra*	90.7	74.7	67.7	57.0
*Faidherbia albida*	98.7	93.7	88.7	87.0

## DISCUSSION

A common way to circumvent the cost and analysis challenges related to whole genome sequencing in large‐scale studies is targeted sequencing. Targeted sequencing can be done via amplicon sequencing or hybridization capture. Amplicon sequencing via highly multiplexed PCR, followed by high‐throughput sequencing, is an easy and cost‐effective way of sequencing thousands of individuals without the need for specialized equipment or sophisticated computational tools (Meek and Larson, 2019). However, the method relies on designing primers flanking the genomic regions of interest to amplify and enrich them for sequencing. This necessitates the availability of genomic information for the species of interest. Hybridization capture, in which high‐throughput sequencing libraries are enriched for regions of interest using capture probes, does not necessarily require the availability of genomic information for the species under study. This is because target capture probes are often designed for in‐group taxa (taxon specific) (e.g., Vatanparast et al., 2018; Soto Gomez et al., 2019) or across taxa (universal) (e.g., Johnson et al., 2019), allowing applicability in a multitude of species. In this study, 11 angiosperm species were used to investigate the presence of sufficient SNPs in the Angiosperms353 target regions. Furthermore, we tested the potential of the identified SNPs for kinship analysis in four of the 11 species with more than one half‐sib family.

### Recovery rate

Angiosperms353 was designed to capture hundreds of putatively orthologous genes from any angiosperm species (Johnson et al., 2019). Even though there was variation within and among species, gene recovery was successful for all individuals, with up to 331 genes per individual having ≥50% of the target length recovered. Different factors, including genomic DNA concentration and quality, library concentration, and pooling strategy, are reported to potentially affect target capture success (Brewer et al., 2019; Andermann et al., 2020). In the present study, no clear relationship between library concentration or genomic DNA concentration and capture efficiency was observed. Another factor that could potentially affect capture efficiency is taxonomic bias. Even though it has been reported that there is little to no taxonomic bias in the sequencing outcome of Angiosperms353 loci (Johnson et al., 2019; Slimp et al., 2021), Brewer et al. (2019) detected bias in some angiosperm families. The species the individuals belonged to appeared in this study to be the most important factor when it comes to capture efficiency, as individuals belonging to the same species tended to have comparable capture efficiencies regardless of the library concentration or the hybridization pool, indicating potential taxonomic bias. However, genomic DNA quality could also be the reason behind the differential performance of species, as different species might have different inhibitors that could affect hybridization success.

### Technical challenges

Although the Angiosperms353 probes target putative single‐copy genes, gene duplication events are frequent in plants (Panchy et al., 2016). Genome duplication has been reported to be a major force behind the evolution of woody angiosperms (Neale et al., 2017). The most‐used analysis pipeline for target enrichment data, HybPiper (Johnson et al., 2016), provides a warning for putatively paralogous genes among the captured ones. However, it is difficult to distinguish orthologs from paralogs due to their high sequence similarity (Altenhoff and Dessimoz, 2019), and the paralog detection in HybPiper may not be 100% efficient (Zhou et al., 2022). Paralogs can cause the collapse of similar sequences originating from different regions into one during assembly, potentially resulting in artificially inflated variants (Ousmael et al., 2023). The presence of excess heterozygotes can indicate mapping issues. In the present study, manually removing loci with paralog warnings did not improve the problem of excess heterozygotes. This indicates that there were either undetected paralogs or that the mapping issue was not limited to the presence of paralogs. The size of the reference used (combined supercontigs) could be another issue, as the aligner finds the best possible alignment for the reads even though the reads may not have originated from the region to which it is aligned (due to the reference missing the duplication, for instance). This phenomenon, referred to as “abusive mapping” in Djedatin et al. (2017), leads to excess heterozygotes. In our study, the global (end‐to‐end) alignment showed a reduced number of false positives and/or heterozygotes compared to the local alignment. To further remove SNPs arising from potential mapping issues, all SNPs with a coverage depth higher than twice the mean depth were removed. An additional step in removing loci with excess heterozygotes was taken in *A*. *glutinosa* and *Q*. *castaneifolia*. Overall, the identification of reliable markers using similar targeted sequencing approaches requires careful consideration in addressing potential challenges related to paralogs and reference bias.

### Variation

The primary objective behind developing the Angiosperms353 probe set was taxonomic analysis, with a focus on highly conserved regions. Its potential application in the kinship analysis of trees is contingent on the availability of sufficient variation within the target region. The minimum number of filtered SNPs identified was 182 in *A*. *velutinum*, with all other species having over 200 SNPs, which is adequate for kinship analysis across all species. Notably, in *A*. *senegal*, despite having only two tetraploid individuals compared to three diploids, the former showed a substantially higher number of SNPs (513 vs. 333). This could be attributed to the increased genetic variation resulting from having more copies of each chromosome, which is potentially influenced by mutations. Previous studies have shown that relatedness analysis can be performed with fewer than 200 SNPs, even in conifers with larger genome sizes (Ousmael et al., 2023). Consistent with this, Flanagan and Jones (2019) stated that a few hundred SNPs are sufficient for parentage analysis. Additionally, Wang and Scribner (2014) reported that even as low as 50 SNPs are generally sufficient for relatedness analysis, except for samples with high relatedness or in cases involving uniformly large, highly related families.

The accurate estimation of pairwise relatedness values requires the use of sufficient samples to estimate allele frequency, which serves as a base for relatedness estimates. As a result, pairwise relatedness values based on a few individuals, as is the case in this study, require careful interpretation. However, the focus of the present study was not to obtain accurate relatedness values but rather to test the potential of finding sufficient SNPs and the ability of those SNPs for the intended purpose. Despite the small number of samples per species, the SNPs identified in the present study resulted in grouping and pairwise relatedness values that were comparable to the expected values based on the available pedigree information in three of the four species with family information. The potential of the identified SNPs was further demonstrated by the relative performance of the SNPs compared to a large panel of DArTseq SNPs in *C*. *africana*, with a very high correlation of pairwise relatedness values (when self, within‐family, and between‐family/provenance relationships are considered) between the two sets of SNPs. The correlation between the relatedness values obtained using Angiosperms353‐based SNPs and DArTseq SNPs appears to be less strong within families. In general, given the small sample size, the relatedness values may not be entirely precise.

The identified SNPs showed better performance in *C*. *africana* and *F*. *albida*, with the two species having a relatively larger number of individuals compared to *A*. *glutinosa* and *Q*. *castaneifolia*. Individuals in *A*. *glutinosa* required additional filtration of the SNPs (removing loci with heterozygosity >0.5 and MAF <0.2) to estimate relatedness values that are close to the expected values. In *Q*. *castaneifolia*, individuals were clustered according to their family; however, despite additional filtration (similar to *A*. *glutinosa*), pairwise relatedness values remained low for most individuals. Although the low level of relatedness between individuals of the families in this species could be due to different factors, it may be attributed to the small number of individuals. Relatedness estimates tend to be biased when allele frequencies are calculated from an extremely small sample size (Wang, 2017). Furthermore, calculating allele frequencies from a sample containing both related and unrelated individuals results in an underestimation of relatedness (Wang, 2017). Ideally, allele frequencies should be estimated from a large reference population of non‐inbred and unrelated individuals, where all homologous genes within and between individuals are not identical by descent (IBD) (Wang, 2014). However, such allele frequencies are rarely available, and in practice, they are often estimated from the genotypes of the sample under study. This necessitates the interpretation of relatedness values as correlations rather than probabilities of IBD, as most relatedness analyses require relative rather than absolute values of relatedness (Wang, 2014). Nevertheless, relatedness in a small sample, estimated using allele frequencies obtained from a small number of related individuals, tends to be underestimated more for related individuals than for unrelated ones (Wang, 2017).

### Potential for scaling up

The application of Angiosperms353 offers the opportunity to identify SNP markers in a single species without a reference genome or in a multitude of species simultaneously, as shown in this study. However, large‐scale applications depend on the utilization of complementary techniques. Two of the potential complementary techniques are multiplexing for hybridization and designing amplicon sequencing primers for the identified markers. Hale et al. (2020) provided several strategies to reduce per‐sample cost in targeted sequencing projects at different stages of the process from DNA extraction to sequencing. One of the strategies was a 3:1 (water:probe) dilution of RNA probes, which, combined with multiplexing of 24 libraries in a quarter of the dilution, allowed hybridization of 96 libraries using probes from a single hybridization reaction. The authors showed that an overall per‐sample cost reduction of more than 50% could be achieved by using their modified workflow. Liu et al. (2019) also demonstrated that 96 libraries can be multiplexed in a single hybridization reaction without affecting target recovery. While this approach is feasible for genotyping up to a few hundred samples, there are additional considerations when the sample size increases to thousands of individuals, as is the case in breeding programs. One issue that needs to be considered is that it is neither necessary nor feasible to sequence the entire target regions at a large enough depth for thousands of individuals if the interest in the context of kinship (relatedness) analysis is simply on the genotype of the individuals at variable sites. Designing amplicon sequencing primers for a selected set of SNPs to create a genotyping panel that can be used to genotype thousands of individuals could be a solution to this problem. We demonstrated the potential of scaling up the method to a large number of individuals by designing primers for selected SNPs and testing their performance in silico, where 85.3% and 87% of the designed primers showed successful in silico amplification in *A*. *glutinosa* and *F*. *albida*, respectively. Another way to validate the primers designed based on the Angiosperms353‐generated sequences is to synthesize them, perform PCR using genomic DNA, and sequence the resulting amplicons. However, we opted for in silico validation (using the reference genomes of the species as a template), which offers a fast and inexpensive alternative.

Through successful validation in *C. africana* and in silico primer testing, we demonstrated the potential use of universal target capture probe‐based SNPs in kinship analysis, showcasing their suitability for large‐scale genotyping in a cost‐effective manner when complemented by amplicon sequencing. This is of particular significance in its application to species lacking genomic resources. Angiosperms353, when coupled with amplicon sequencing, emerges as a valuable tool for large‐scale genotyping and relatedness analysis in these species. This is crucial for enhancing our understanding of population structure and individual relatedness, especially in the context of breeding initiatives that require large‐scale genotyping, and is particularly valuable for breeding programs based on pedigree reconstruction.

### Examples of applications

Small SNP genotyping sets (ranging from a few dozen to a few hundred SNPs) are invaluable tools in various plant science and agricultural applications, including pedigree reconstruction, which enables researchers to accurately trace or verify lineages (Lukman et al., 2014; Flanagan and Jones, 2019; Akpertey et al., 2021), and purity testing, including clone and identity analysis, which confirms the genetic purity and authenticity of plant materials. For example, Ousmael et al. (2023) were able to identify clones and mislabeled individuals in the non‐model conifer *Abies nordmanniana* (Steven) Spach using only 50 SNPs. Additionally, small genotyping sets can be used for genetic diversity assessment (Foster et al., 2010; Akpertey et al., 2021), helping to evaluate and maintain genetic variation within and between plant populations. These applications are essential for effective breeding, conservation, and utilization.

### Limitations

The performance of the SNPs in *A*. *glutinosa* and *Q*. *castaneifolia* relative to the performance in *C*. *africana* and *F*. *albida* suggests that using more individuals (ideally more than two families per species) might improve marker performance. However, if the intention is to identify SNPs that will be used to genotype a large sample population, as is the context of this study, the bias in relatedness analysis might not be of great concern at the marker development stage.

The identification of SNPs within the Angiosperms353 target regions is dependent on successful target recovery, a factor that cannot be guaranteed due to the high variation observed in target recovery among species. In our study, the species with the lowest target recovery (*C*. *africana*), which also exhibited the second lowest number of filtered SNPs (237), allowed for the resolution of relatedness in a manner that correlated 95% with the results obtained from thousands of genome‐wide SNPs. However, for studies requiring a substantial number of SNPs and comprehensive genome‐wide sampling, alternative SNP identification methods are more appropriate. The applicability in gymnosperms should be tested using appropriate target capture probes, such as REMcon (Khan et al., 2024).

## CONCLUSIONS

Our study highlights the potential of the Angiosperms353 probe set for the development of SNPs in different angiosperm species lacking genomic information. The variation within the target regions, as evidenced by over 300 SNPs in most species, supports the utility of Angiosperms353‐based SNPs for kinship as well as other population genetic analyses. Furthermore, our results demonstrate the scalability of the approach through the successful design and in silico testing of primers for selected SNPs. The combination of Angiosperms353 and amplicon sequencing can be used as a cost‐effective solution for large‐scale genotyping, which is particularly valuable for breeding programs based on pedigree reconstruction.

## AUTHOR CONTRIBUTIONS

K.M.O. and O.K.H. conceptualized the study. K.M.O. designed the study. K.M.O. and O.K.H. collected samples. K.M.O. extracted DNA. K.M.O. analyzed the data with supervision from O.K.H. K.M.O. wrote the first draft and O.K.H. contributed to development of the paper through methodological advice, reviews, and edits of the text. Both authors approved the final version of the manuscript.

## Supporting information


**Appendix S1**. Sample information, Qubit quantification values in DNA extracts and libraries, bait capture pooling scheme, and total number of raw reads from paired‐end sequencing.

**Appendix S2**. Target (supercontig) recovery statistics.

**Appendix S3**. Minimum, maximum, and mean individual single gene supercontig length.

## Data Availability

The targeted sequencing reads, recovered supercontigs, and reference sequences generated and/or analyzed during this study are publicly available in the University of Copenhagen Electronic Research Data Archive (ERDA): https://doi.org/10.17894/ucph.fea7386b-099c-4655-bf8d-d84fa681a6b6.
